# Automatic Rate and Rate-Integrating Mode-Switchable Axisymmetric Gyroscope

**DOI:** 10.3390/s22124334

**Published:** 2022-06-08

**Authors:** Lin Xuan, Mingyang Lu, Jing Liu, Shuwen Guo, Dacheng Xu

**Affiliations:** School of Electronic and Information Engineering, Soochow University, Suzhou 251000, China; 20194228005@stu.suda.edu.cn (L.X.); 20195228066@stu.suda.edu.cn (M.L.); liujing_syerll@163.com (J.L.); 13862044796@163.com (S.G.)

**Keywords:** MEMS gyroscope, mode switching, rate mode, rate-integrating mode

## Abstract

This paper describes a novel rate and rate-integrating mode-switchable axisymmetric gyroscope. A precession angle tracking algorithm is developed to enable the gyro to switch automatically between rate and rate-integrating modes at preset rate points through a digital control system within the gyro. We also propose a vibrating amplitude control method for the rate-integrating mode that directly extracts the angular rate output to ensure switching stability. In rate mode, the bias instability and angle random walk of the gyro reach 0.106°/h and 0.011°/√h, respectively. Additionally, an input range of over ±5000°/s is measured in rate-integrating mode. The scale factor nonlinearity reaches approximately 116 ppm over the full-scale range. The control system implements effective steering control of the gyroscope, with a switching delay of 10 ms from rate mode to rate-integrating mode and 100 ms from rate-integrating to rate mode. The proposed system actualizes a new type of gyroscope with high accuracy and a wide input range, which combines the benefits of rate and rate-integrating modes.

## 1. Introduction

Silicon micro gyroscopes are widely used in the automotive, consumer electronics, industrial, and aerospace fields due to their small size, low power consumption, and low cost [1]. Axisymmetric gyroscopes can be categorized as either rate gyroscopes (RGs) or rate-integrating gyroscopes (RIGs), depending on whether they measure angular rates or rotation angles [2,3,4,5,6]. RG operating in force-to-rebalance mode applies a feedback force that cancels the Coriolis force and detects the angular rate by measuring the magnitude of the feedback force. This approach is highly accurate, but the size of the supply voltage limits the range of this mode. RIG mode, also called whole-angle mode, is an operational mode in which the Coriolis force causes the oscillation pattern of the gyro to precess freely, and the input angle is detected by measuring the change in the precession angle of the resonant modes. Theoretically, RIG mode has unlimited bandwidth and range as well as high stability of the angular gain [7,8,9]. However, manufacturing errors can result in damping asymmetry and stiffness asymmetry [10], leading to a minimum rate threshold for RIGs [11,12]. The RIG mode cannot operate at rates below this threshold, but it is suitable for higher rates.

The research focus of RGs and RIGs is on improving accuracy and reducing the threshold, respectively. The accuracy of RGs can be improved by using electrostatic tuning and quadrature closed-loop control to reduce the frequency split (Δ*f*) and quadrature errors [13,14,15]. However, the magnitude of the feedback force in the circuit is limited by the operating voltage, making it difficult to achieve a large range for RGs;in the abovementioned references, the range does not exceed ±300°/s. For RIGs, the coarse compensation for the quadrature electrostatic tuning and frequency electrostatic tuning as well as quadrature closed-loop control can only eliminate quadrature errors, so the residual error still has a large impact on the minimum rate threshold. A damping anisotropy estimation model [16] compensates the damping error, reducing its impact by 25%. References [11,12,17] use virtual rotation by applying a fixed force to the precession angle control loop, thus reducing the threshold and angle-dependent bias due to damping asymmetry. Thus, the oscillation mode of the gyro can rotate at a constant rate without the angular rate being input. However, this method is limited by the hardware circuit, and the angle generated by the virtual rotation must be subtracted, which increases the difficulty of real-time observation. Reference [18] describes a combined virtual rotation and damping error model that allows the RIG threshold to be reduced to 1°/s. The method adopted in [19] analyzes the error terms in the precession angular rate and compensates the stiffness asymmetry and damping asymmetry based on Fourier series fitting combined with a multiple iteration technique, resulting in a RIG threshold of 0.05°/s. The methods described above provide some suppression of damping and stiffness errors, but several issues require further improvement, such as the complex implementation circuits and experimental methods. Reference [20] indirectly augments the RG measurement range, and it reduces the RIG threshold by switching between operational modes, that is, the gyro operates in RG mode for low rates and when high-precision measurements are required, and it switches to RIG mode when high rates and wide-range measurements are required. This method avoids the need for complex circuits and implementation protocols, and it effectively takes advantage of the high precision offered by RGs and the wide-range measurements achieved by RIGs. Reference [21] mentions the use of a computer GUI to send a command to the digital board to control the switching between operational modes. Unfortunately, neither [20] nor [21] provides detailed implementation details of the mode switching process. Additionally, [20] only shows the experimental results of the process of switching from RG to RIG, and the angular output in RIG mode differs from the angular rate output in RG mode, which makes the outputs inconsistent over a full-scale range.

In this paper, we describe a control system for switching between RIG and RG modes, and we propose an angular rate output method in RIG mode to obtain the angular rate output, which can achieve a uniform rate output in two operation modes under full-scale range conditions. Additionally, we describe how automatic mode switching can be achieved in real-time operations. The switching control system automatically switches between RG and RIG modes through angle open-loop or closed-loop control according to the preset switching threshold and a precession angle tracking algorithm. The system adopts an RG mode at low rates and automatically switches to RIG mode at high rates, combining the advantages of both modes to give a microelectromechanical systems (MEMS) axisymmetric gyro, with high accuracy and a wide input range.

## 2. Theory

### 2.1. Dynamic Equation

**Figure 1:**
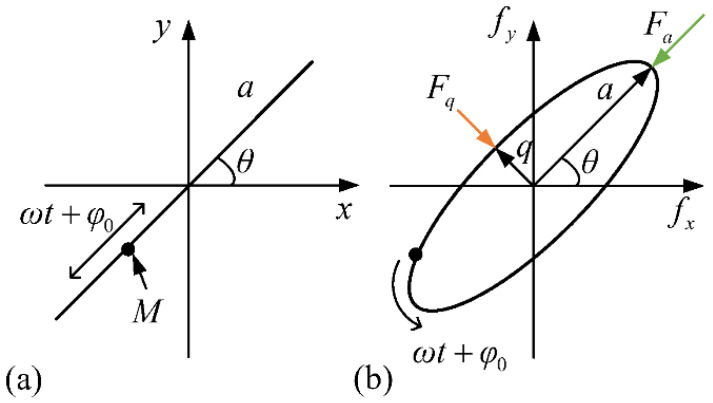
(**a**) Ideal and (**b**) non-ideal vibration patterns of the gyro.

The dynamics of axisymmetric gyroscopes are generally in a non-ideal state due to processing and material factors, which cause problems such as damping and stiffness asymmetries. The dynamic equations of axisymmetric gyroscopes can be written as [22]:

(1) $$\begin{array}{l} \left[\begin{array}{c} \ddot{x}\\ \ddot{y} \end{array}\right]+\left[\begin{array}{cc} \left(\frac{2}{\tau }\right)+\left(\Delta \frac{1}{\tau }\right)\cos2\theta_{\tau } & \left(\Delta \frac{1}{\tau }\right)\sin2\theta_{\tau }\\ \left(\Delta \frac{1}{\tau }\right)\sin2\theta_{\tau } & \left(\frac{2}{\tau }\right)-\left(\Delta \frac{1}{\tau }\right)\cos2\theta_{\tau } \end{array}\right]\left[\begin{array}{c} \dot{x}\\ \dot{y} \end{array}\right]\\ +\left[\begin{array}{cc} \omega^{2}+\omega \Delta \omega \cos2\theta_{\omega } & \omega \Delta \omega \sin2\theta_{\omega }\\ \omega \Delta \omega \sin2\theta_{\omega } & \omega^{2}-\omega \Delta \omega \cos2\theta_{\omega } \end{array}\right]\left[\begin{array}{c} x\\ y \end{array}\right]+\left[\begin{array}{cc} 0 & -2nk\Omega \\ 2nk\Omega & 0 \end{array}\right]\left[\begin{array}{c} \dot{x}\\ \dot{y} \end{array}\right]=\frac{1}{M}\left[\begin{array}{c} F_{x}\\ F_{y} \end{array}\right] \end{array}$$

where *x* and *y* are the vibration amplitudes of two resonant modes, $\dot{x}$, $\dot{y}$ are the velocities of *x*, *y*, and $\ddot{x}$, $\ddot{y}$ are the accelerations of *x*, *y*; Δ*ω* and *ω* are, respectively, associated with the difference and the average of the resonant frequencies of the two modes; 1/*τ* is the damping coefficient; Δ(1/*τ*) is the damping error; *θ_τ_*, *θ_ω_* are the included angles between the damping principal axis and the stiffness principal axis, respectively; *k* is the angular gain; *n* is the order of vibration modes [23]; Ω is the input angular rate; *M* is the effective mass of the gyro; and *F_x_*, *F_y_* are the forces in the *x*- and *y*-directions. From the point of view of vibration equations, the particle motion trajectory of the gyro can be regarded as an ellipse, as shown in Figure 1b.

The vibration amplitudes can be written as:

(2) $$\begin{array}{l} x=a\cos\theta \cos\left(\omega t+\varphi \right)-q\sin\theta \sin\left(\omega t+\varphi \right)\\ y=a\sin\theta \cos\left(\omega t+\varphi \right)+q\cos\theta \sin\left(\omega t+\varphi \right) \end{array}$$

where *a* is the amplitude of oscillation, *q* is the quadrature motion, *θ* is the precession angle of the ellipse motion of vibration, and *φ* is the phase of the oscillation.

The control forces along the *x*- and *y*-axes can be obtained as follows:

(3) $$\begin{array}{l} F_{x}=F_{a}\cos\theta -F_{q}\sin\theta \\ F_{y}=F_{a}\sin\theta +F_{q}\cos\theta \end{array}$$

in which

(4) $$\begin{array}{l} F_{a}=F_{ac}\cos\omega t+F_{as}\sin\omega t\\ F_{q}=F_{qc}\cos\omega t+F_{qs}\sin\omega t \end{array}$$

In (4), *F_as_* and *F_ac_* are the sine and cosine components of *F_a_*, respectively, *F_qs_* and *F_qc_* are the sine and cosine components of *F_q_*, respectively. We can obtain the ellipse parameters by averaging the equations of motion in Equation (1) over a period of vibration [24]:

(5) $$\begin{array}{l} \dot{a}=-\frac{q}{2}\Delta \omega \sin2\left(\theta -\theta_{\omega }\right)-a\left(\frac{1}{\tau }+\frac{1}{2}\Delta \frac{1}{\tau }\cos2\left(\theta -\theta_{\tau }\right)\right)-\frac{F_{as}}{2\omega M}\\ \dot{q}=-\frac{q}{2}\left(\frac{1}{\tau }-\frac{1}{2}\Delta \frac{1}{\tau }\cos2\left(\theta -\theta_{\tau }\right)\right)+\frac{a}{2}\Delta \omega \sin2\left(\theta -\theta_{\omega }\right)+\frac{F_{qc}}{2\omega M}\\ \dot{\theta }=-\frac{aq}{a^{2}-q^{2}}\Delta \omega \cos2\left(\theta -\theta_{\omega }\right)+\frac{1}{2}\frac{a^{2}+q^{2}}{a^{2}-q^{2}}\Delta \frac{1}{\tau }\sin2\left(\theta -\theta_{\tau }\right)-k\Omega +\frac{qF_{ac}-aF_{qs}}{2\omega M\left(a^{2}-q^{2}\right)}\\ \dot{\varphi }=\frac{1}{2}\frac{a^{2}+q^{2}}{a^{2}-q^{2}}\Delta \omega \cos2\left(\theta -\theta_{\omega }\right)-\frac{aq}{a^{2}-q^{2}}\Delta \frac{1}{\tau }\sin2\left(\theta -\theta_{\tau }\right)-\frac{aF_{ac}-qF_{qs}}{2\omega M\left(a^{2}-q^{2}\right)} \end{array}$$

In (5), *a*, *q*, *θ* can be controlled by *F_as_*, *F_qc_*, *F_qs_*, respectively. The motion parameters are all affected by damping asymmetry and stiffness asymmetry.

### 2.2. Control Algorithm

According to Lynch’s algorithm [25], the vibration amplitudes can be demodulated by multiplication to obtain the following four vibration pattern parameters:

(6) $$\begin{array}{l} x_{c}=a\cos\theta \cos\varphi +q\sin\theta \sin\varphi \\ x_{s}=a\cos\theta \sin\varphi -q\sin\theta \cos\varphi \\ y_{c}=a\sin\theta \cos\varphi -q\cos\theta \sin\varphi \\ y_{s}=a\sin\theta \sin\varphi +q\cos\theta \cos\varphi \end{array}$$

The control variables for the energy (*E*), quadrature (*Q*), precession angle (*θ*), and phase reference error (*φ*) can be obtained by applying a combination operation:

(7) $$\begin{array}{l} E=a^{2}+q^{2}=x_{c}^{2}+x_{s}^{2}+y_{c}^{2}+y_{s}^{2}\\ Q=2aq=2(x_{c}y_{s}-x_{s}y_{c})\\ R=\left(a^{2}-q^{2}\right)\cos2\theta =x_{c}^{2}+x_{s}^{2}-y_{c}^{2}-y_{s}^{2}\\ S=\left(a^{2}-q^{2}\right)\sin2\theta =2\left(x_{c}y_{c}+x_{s}y_{s}\right)\\ \theta =\frac{1}{2}\arctan\left(\frac{S}{R}\right)\\ \varphi =\frac{1}{2}\arctan\frac{2\left(x_{c}x_{s}+y_{c}y_{s}\right)}{x_{c}^{2}-x_{s}^{2}+y_{c}^{2}-y_{s}^{2}} \end{array}$$

where *θ* can be used for angle control, which allows the oscillation pattern to be fixed at the reference value *θ*_0_ in RG operation mode; *φ* is controlled by a phase-locked loop (PLL) to track the resonant frequency of oscillation. The variables *E*, *Q*, and *θ* form energy, quadrature, and angle control loops, respectively, through proportional-integral (PI) controllers, which are used to maintain the vibration amplitude, reduce the quadrature error, and fix the precession angle, respectively. The corresponding outputs of PI controllers *F_E_*, *F_Q_*, and *F_θ_* can be described as:

(8) $$\begin{array}{l} F_{E}=F_{as}=K_{PE}\left(E-E_{0}\right)+K_{IE}\int_{0}^{t}\left(E\left(\tau \right)-E_{0}\right)d\tau \\ F_{Q}=F_{qc}=-K_{PQ}Q-K_{IQ}\int_{0}^{t}Q\left(\tau \right)d\tau \\ F_{\theta }=F_{qs}=K_{PS}\left(\theta -\theta_{0}\right)-K_{IS}\int_{0}^{t}\left(\theta \left(\tau \right)-\theta_{0}\right)d\tau \end{array}$$

where *K_P_* and *K_I_* are PI parameters and *E*_0_ is the reference value for the energy loop. The feedback forces *F_x_* and *F_y_* can then be derived as:

(9) $$\begin{array}{l} F_{x}=F_{E}\cos\theta \sin\omega t-F_{Q}\sin\theta \cos\omega t-F_{\theta }\sin\theta \sin\omega t\\ F_{y}=F_{E}\sin\theta \sin\omega t+F_{Q}\cos\theta \cos\omega t+F_{\theta }\cos\theta \sin\omega t \end{array}$$

The open-loop angle control enables the gyro to operate in RIG mode with a freely-moving oscillation pattern regulated by the Coriolis force, that is *F_θ_* = 0. The closed-loop angle control forces the gyro to operate in RG mode by fixing the precession angle, the PI controller output *F_θ_* reflects the detected angular rate. Therefore, the control algorithm enables the gyro to operate in RIG or RG mode, and it realizes the switching between the two modes through the angle open-loop/closed-loop control, providing a theoretical basis for the switching system.

### 2.3. Angular Rate Output in RIG Mode

Considering a detected system cannot identify the high or the low rotation rates with angle information, it is necessary to obtain the angular rate information of the RIG mode in real time for switching from RIG to RG mode. In RIG mode, the force applied in the angle control open-loop scheme is used to control the direction of the oscillation [26]. Additionally, the Coriolis force arising from the rotation causes the oscillation pattern to freely precess, indicating that the input angular rate can be detected by measuring the Coriolis force in the angle control loop. As mentioned in [27], the force containing angular rate information can be extracted through the demodulation of vibration amplitudes without considering quadrature errors, but the underlying expressions for this operation have not yet been derived, due to their work mainly being focused on using other forces to compensate for the relevant errors. In RIG mode, in addition to the change in the precession angle (from which we obtain the angle output), the angular rate can be obtained through a demodulation and a combinatorial calculation of amplitudes of two resonance modes. Therefore, we independently derived the theoretical equation for the rate output in RIG mode, and we performed a successful experimental verification of the equation.

In the absence of the quadrature error and input angular rates, Equation (2) can be described as:

(10) $$\begin{array}{l} x=a\cos\theta_{0}\cos\omega t\\ y=asin\theta_{0}\cos\omega t \end{array}$$

**Figure 2:**
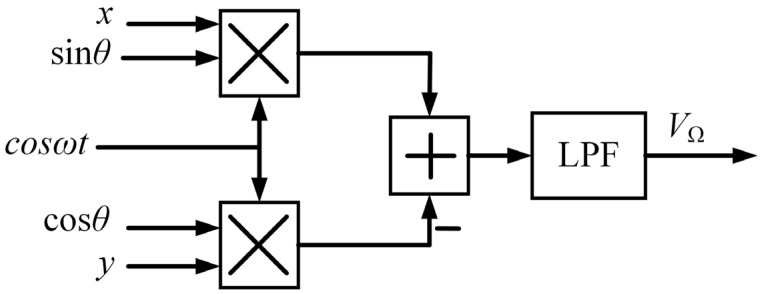
Block diagram of obtaining the detected angular rate *V*_Ω_.

The Coriolis forces of the *x*- and *y*-axes *F_cx_*, *F_cy_* in RIG mode can be written as:

(11) $$\begin{array}{l} F_{cx}=-F_{c}\sin\theta \sin\omega t\\ F_{cy}=F_{c}\cos\theta \sin\omega t \end{array}$$

where *F_c_* is the amplitude of the Coriolis force, which is proportional to the input angular rate Ω. When Ω is input, the forces from (11) produce the phase-shifted displacements after passing through the gyro, and the displacement changes accordingly:

(12) $$\begin{array}{l} x=a\cos\theta \cos\omega t-\frac{k_{fd}F_{c}}{M}\sin\theta \cos\omega t\\ y=asin\theta \cos\omega t+\frac{k_{fd}F_{c}}{M}\cos\theta \cos\omega t \end{array}$$

where *k_fd_* is the force-to-displacement coefficient. Equation (12) indicates that Ω influences the amplitude of two resonate modes, and it has a considerable effect at larger rates. The detected angular rate *V*_Ω_ can be calculated as:

(13) $$V_{\Omega }=2\left(x\sin\theta \cos\omega t-y\cos\theta \cos\omega t\right)k_{e}\overset{LPF}{=}\frac{k_{fd}k_{e}F_{c}}{M}\propto -k\Omega$$

where *k_e_* is the gain of the hardware circuit. The rate signal *V*_Ω_ is proportional to Ω, and it is related to *M*, *k_fd_*, and *k_e_*. Based on Equation (13), the calculation block diagram can be described in Figure 2.

**Figure 3:**
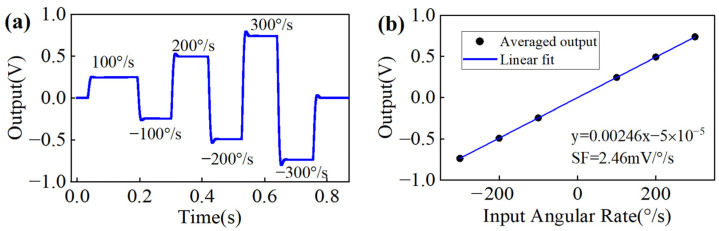
Simulation results for (**a**) angular rate output in RIG mode and (**b**) scale factor fitting curve.

Figure 3a shows the simulation results for the angular rate directly output in RIG mode by a vibrating amplitude control method, where the input angular rates are ±100°/s, ±200°/s, and ±300°/s. The simulation parameters are listed in Table 1. Figure 3b shows that the average output is proportional to the input angular rate and that the scale factor is approximately 2.46 mV/°/s. The simulation and the measurement results prove the correctness of (13) and provide a basis for improving the switching stability.

### 2.4. Switching Control

**Figure 4:**
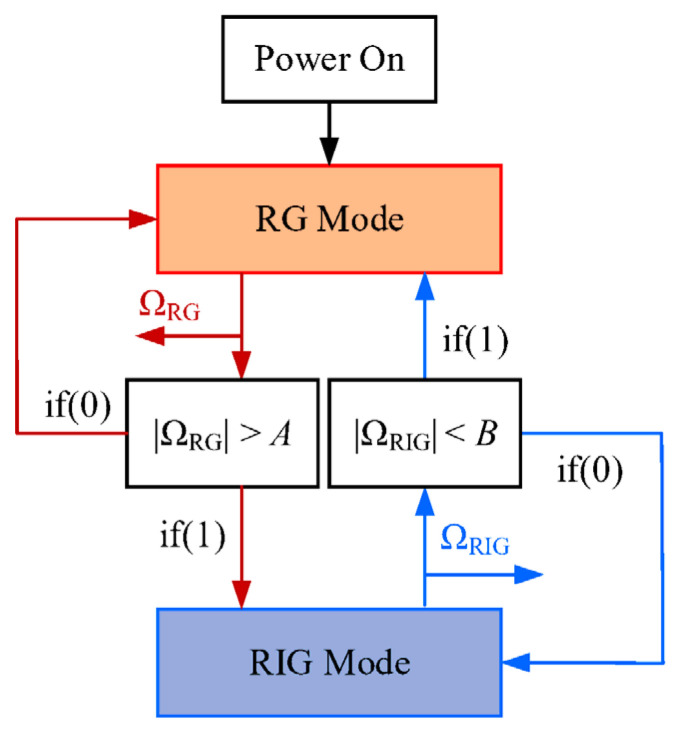
Concept of switching between RG and RIG modes.

To ensure that mode switching is carried out reliably and automatically, we choose different preset rate points for the two operational modes, where *A* and *B* are the preset RG-to-RIG and RIG-to-RG thresholds, respectively. Ω_RG_ and Ω_RIG_ are the rate outputs of RG and RIG modes, respectively, as shown in Figure 4. We can set switching thresholds as required for the condition *A* > *B*, as the system can misjudge or make repeated switches when *A* ≤ *B*, resulting in unstable or failed switches. The switch module consists of three functions for automatic comparison, switching, and precession angle tracking (Figure 4). The gyro operates in RG mode when powered on and switches to RIG mode with open-loop angle control when the output satisfies $\left|\Omega_{RG}\right|>A$. In RIG mode, the system tracks the precession angle information in real time. When $\left|\Omega_{RIG}\right|<B$ is satisfied, the information stops refreshing, and it is used as the reference value for the closed-loop angle control to switch back to RG mode.

**Figure 5:**
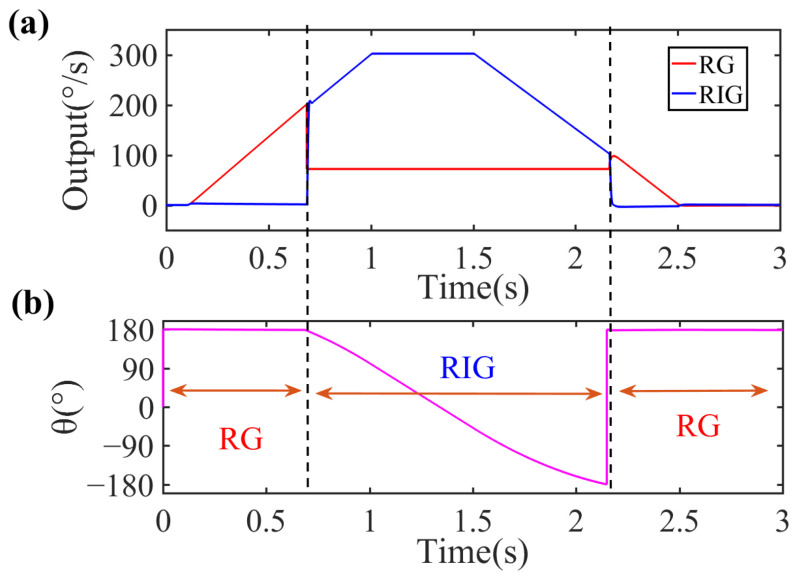
(**a**) Angular rate and (**b**) precession angle simulation results during automatic mode switching.

Figure 5 shows the simulated rate and the angle outputs of the automatic mode switching. The output signals in Figure 4 can be divided into three stages. In the first stage, the gyro operates in RG mode with a fixed precession angle. As the input rate accelerates in the second stage, the gyro switches from RG mode to RIG mode and the precession angle begins to change when the input rate is above 200°/s. In the third stage, the gyro switches back to RG mode and the precession angle becomes constant again when the input rate is less than 100°/s. The RG and RIG outputs are expressed in terms of angular rate by dividing the output voltage by the scale factor. The simulation implements mode switching for the gyro operated in RG mode when under low rates and in RIG mode when under high rates.

## 3. Experimental Results and Discussion

### 3.1. Device and Test Platform

**Figure 6:**
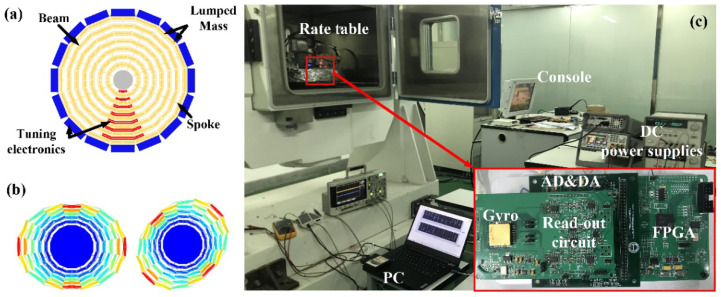
(**a**) Structure and (**b**) mode shapes of the *n* = 2 wineglass modes of the CDRG. (**c**) Test platform and control circuit. The control circuit includes the interface circuit to extract gyro signals, an ADC, and a DAC for the conversion of digital and analog signals and an FPGA to realize the control program.

**Figure 7:**
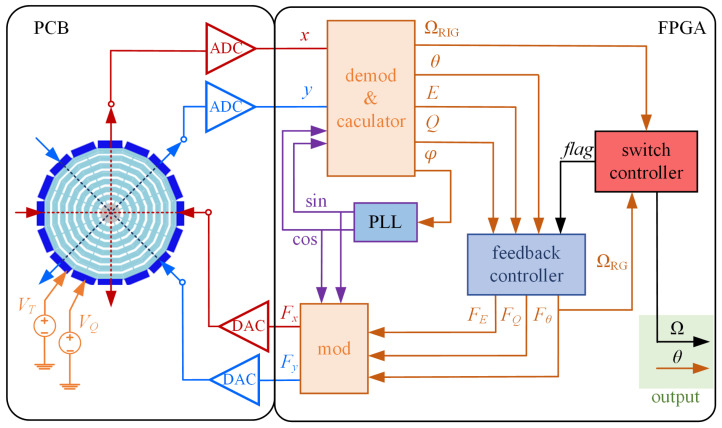
Block diagram of mode switching control system. The ‘demod & calculator’ module in the FPGA is used to demodulate and calculate the required control signals; the PLL module is used to track the resonant frequency of the gyro; the switch controller module is used to compare the angular rate and the preset rate and send the switching control *flag*; and the feedback controller and ‘mod’ modules are used to generate and to modulate the feedback signals to the gyro.

A cobweb-like disk resonator gyroscope (CDRG) [28] was used in the experiments. Figure 6a,b shows the structure and the mode shapes of the CDRG. The CDRG is composed of 10 concentric polygonal spider web rings connected by eight spokes to a single central anchor. Each ring is connected by 16 identical rectangular beams. Table 2 presents the main structural parameters of the CDRG. Figure 6c shows the test platform and control circuit. Figure 7 shows a block diagram of the mode switching control system.

### 3.2. Scale Factor

The DC voltages *V_Q_* and *V_T_* are applied in corresponding electrodes to the anisostiffness compensation for a CDRG. The raw output of PI controller *F_θ_* is used as the rate data in RG mode. The RIG’s rate output is measured by (13).

**Figure 8:**
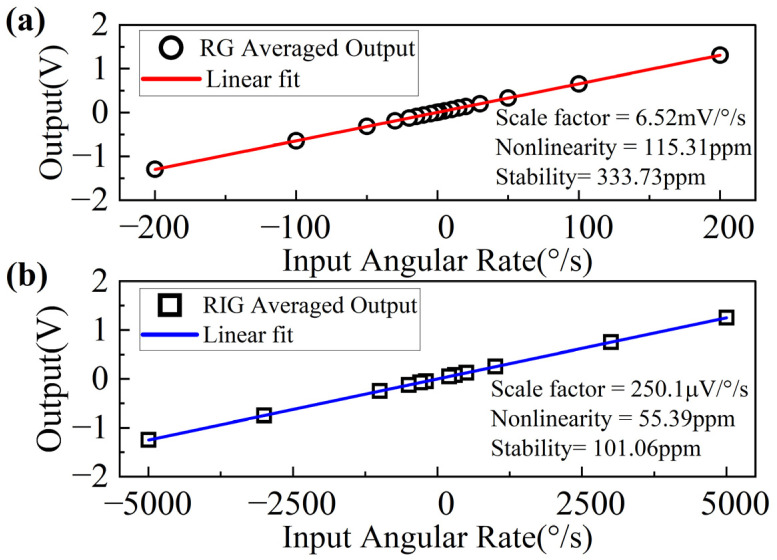
(**a**) RG and (**b**) RIG model scale factor fitting curves.

Scale factor tests were carried out on a rate table at room temperature. The gyro outputs were measured under rotation rates of ±0.1°/s, ±1°/s, ±5°/s, ±10°/s, ±15°/s, ±20°/s, ±30°/s, ±50°/s, ±100°/s, ±200°/s in RG mode and ±200°/s, ±300°/s, ±500°/s, ±1000°/s, ±3000°/s, ±5000°/s in RIG mode. Figure 8a,b shows the scale factor fitting curves in RG and RIG modes, respectively. The measured value is 6.52 mV/°/s in RG mode and 250.1 μV/°/s in RIG mode. The scale factor nonlinearity of the RG and RIG modes in their measurement ranges reaches approximately 116 ppm and 56 ppm, respectively, and the scale factor stability is 334 ppm and 102 ppm, respectively. The RIG operation mode achieves better scale factor performance at larger rates.

### 3.3. Zero-Rate Output Performance in RG Mode

**Figure 9:**
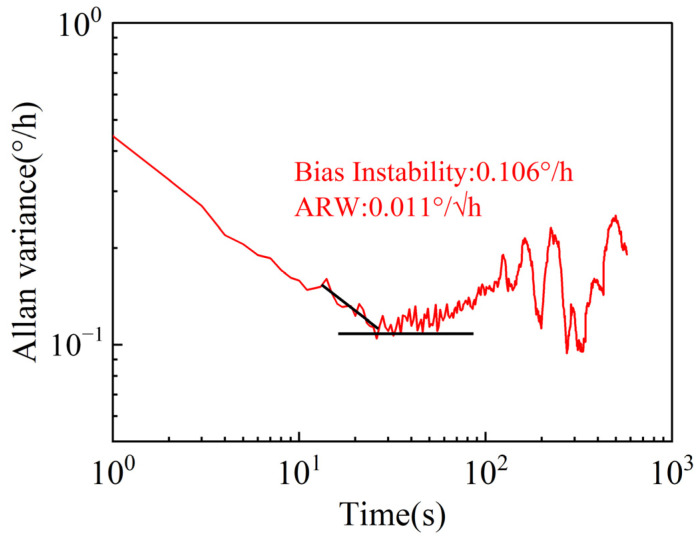
Allan variance curve.

After the open-loop mode-matching operation, zero-rate output (ZRO) data from the FPGA were collected through the serial port at room temperature over an acquisition time of more than 30 min and a sampling frequency of 1 Hz. Figure 9 shows the Allan variance curve of the ZRO data. The figure shows that the bias instability (BI) and angle random walk (ARW) are 0.106°/h and 0.011°/√h, respectively.

### 3.4. Mode Switching

**Figure 10:**
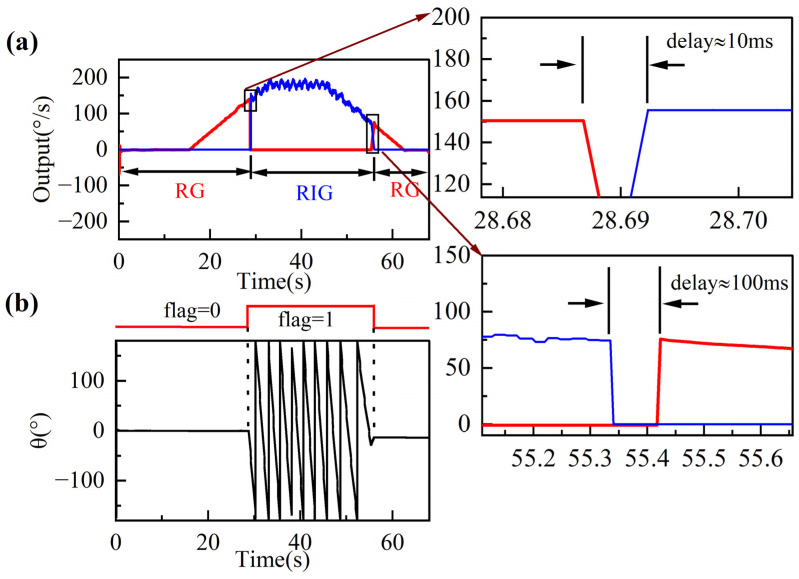
Process of mode switching. (**a**) Angular rate outputs of the gyro and (**b**) corresponding precession angle. The gyro starts in RG mode and switches to RIG mode after a delay of about 10 ms when the input is greater than 150°/s. It switches back to RG mode after a delay of about 100 ms when the input is less than 75°/s. The angle remains fixed in RG mode and precesses freely in RIG mode.

According to the experimental scale factor results, the maximum nonlinearity of the RG’s scale factor occurs at 200°/s. Thus, we set the thresholds to *A* = 150°/s and *B* = 75°/s based on the *A* > *B* condition. Figure 10 shows the experimental results of the mode switching and the corresponding precession angle output. In Figure 10a, the red lines represent the rate output in RG mode, whereas the blue lines represent the measured rate in RIG mode. The gyro starts in RG mode and the vibration pattern is fixed at 0°. When the input angular rate accelerates beyond 150°/s, the switching control input *flag* is set to the high level, the gyro switches to RIG mode, where the vibration pattern starts to precess freely. As the input decelerates below 75°/s, the *flag* is reset to the low level, the gyro switches to RG mode, and the vibration pattern becomes fixed again. As the scale factors of the RG and RIG are not equal, their outputs are expressed as the measured voltage divided by their scale factors in degrees per second. The switching error from RG mode to RIG mode is about ±1°/s with a switching delay of 10 ms, compared with about ±4°/s and 100 ms from RIG mode to RG mode. The switching delay is the time from when the output of the former mode stops to when the output of the latter mode becomes stable. The switching system has a measurement range of ±0–150°/s in RG mode and ±75–5000°/s in RIG mode, although the two operation modes do not work simultaneously.

Figure 10a also shows that the control system switches between RG and RIG modes successfully and automatically. The rate output of the RIG (blue lines) still has an angle-dependent bias error during rotation due to residual asymmetric errors as clearly demonstrated in (5) and (11). However, in this paper, the gyro only operates in RIG mode at high rates, so the angle-dependent bias has less impact on the performance of the RIG in this situation.

Because the RG and RIG modes output the angular rate in different ways, there will be breakpoints in the rate data after switching, and the number of breakpoints is related to the switching delay. The RG-to-RIG delay can be further reduced by program optimization in FPGA. The RIG-to-RG delay can be further improved by optimizing the PI parameters. The bandwidth of the CDRG will be presented as the bandwidth of RG mode if the input angular rate is less than the switching threshold *A*, whereas the bandwidth of the CDRG will be limited by the switching delay if the input angular rate is greater than *A*. Figure 10b shows that the *flag* matches the characteristics of angle output of each operating mode. RIG mode can output both an angular rate and an angle measurement, allowing for full-scale angle and rate detection.

## 4. Conclusions

With the continuous improvement of axisymmetric MEMS gyroscopes, their applications are advancing towards the navigation level and a wide measurement range. This paper has described a switching control system that takes advantage of the high accuracy of RGs and the wide input range of RIGs. In RIG mode, we present the successful simulating and the experimental verification of the algorithm to extract the output angular rate by demodulating and calculating the vibration amplitudes, thus providing a new method of rate detection without the angle derivation operation. The automatic switching between RG/RIG modes is achieved by presetting the switching rate point and controlling the switching *flag*. The RIG-to-RG switching error is ±4°/s and the switching delay is 100 ms. The RG-to-RIG switching error is ±1°/s and the switching delay is 10 ms. The gyro with the proposed system has a BI of 0.106°/h, an ARW of 0.011°/√h, and an input range of over ±5000°/s, achieving both a high accuracy and a large range of MEMS axisymmetric gyros.

On this basis, through further optimization of the error model, control program, and self-compensation techniques, it should be possible to improve the output accuracy of the RG and to reduce the threshold of the RIG and the delays of switching, laying the foundation for broadening the application range of silicon MEMS axisymmetric gyros. Moreover, the proposed system and method can be applied to other axially symmetric gyros, such as hemispherical gyros.

## Figures and Tables

**Table 1:** Simulation parameters related to rig operation mode.

Parameters	Value	Units
*f_c_* ^1^	5045	Hz
*k*	1	\
Δ*f*	0	Hz
Δ(1/*τ*)	0	rad
√*E*	0.2	μm
*θ_ω_*	0	°
*θ_τ_*	0	°

^1^ *f_c_* = 2π*ω*.

**Table 2:** Main parameters of the CDRG.

Parameters	Value	Units
*f_c_*	5045.93	Hz
*Δf*	1.66	Hz
*Qx*	136,252	\
*Q_y_*	135,606	\
Δ(1/*τ*)	5.35 × 10^−4^	rad
*k*	0.77	\
*M*	4.5 × 10^−7^	kg

## Data Availability

Not applicable.
